# Lignocellulosic Biomass Fractionation by Mineral Acids and Resulting Extract Purification Processes: Conditions, Yields, and Purities

**DOI:** 10.3390/molecules24234273

**Published:** 2019-11-23

**Authors:** Vincent Oriez, Jérôme Peydecastaing, Pierre-Yves Pontalier

**Affiliations:** Laboratoire de Chimie Agro-industrielle (LCA), Université de Toulouse, INRA, INPT, 4 allée Emile Monso, 31030 Toulouse, France; jerome.peydecastaing@ensiacet.fr

**Keywords:** lignocellulose, acid fractionation, monomeric sugars, extract purification, yield and purity

## Abstract

Fractionation of lignocellulose is a fundamental step in the valorization of cellulose, hemicelluloses, and lignin to produce various sustainable fuels and chemicals. Mineral acid fractionation is one of the most applied process and leads to the solubilization and hydrolysis of cellulose and hemicelluloses, whereas most of the lignin remains insoluble and can be separated from the extract. The obtained monomeric sugars in the acid extract are in solution with salts, sugar degradation products, and phenolic molecules. Downstream processing is required to purify the sugars and further valorize them into fuels or chemicals with the use of chemical or biochemical reactions. This purification step also allows the recycling of the mineral acid and the valorization of the sugar degradation products and the co-extracted phenolic molecules, adding value to the whole biorefinery scheme. Many purification techniques have been studied, providing several options in terms of yields, purities, and cost of the process. This review presents the conditions used for the mineral acid fractionation step and a wide variety of purification techniques applied on the obtained hydrolysate, with a focus on the associated yields and purities. Values from the literature are expressed in a standard way in order to simplify comparison between the different processes.

## 1. Introduction

In second-generation biorefineries, agricultural by-products or forest biomass are processed to produce energy and a wide variety of precursor chemicals and bio-based materials, similar to the modern petroleum refineries. Among the variety of possible products manufactured in second-generation biorefineries, liquid transportation fuels mainly in the form of ethanol have been rapidly gaining significance [1].

Lignocellulosic materials do not contain monosaccharides readily available for bioconversion. First, they contain polysaccharides—cellulose and hemicelluloses—which have to be hydrolysed, by means of acids or enzymes, to fermentable sugars. Secondly, cellulose in plants is closely associated with hemicelluloses and lignin, preventing the access of hydrolytic agents to cellulose. Thirdly, the crystalline structure of cellulose itself represents an extra obstacle to hydrolysis. Therefore, a pretreatment is required to remove the lignin and hemicelluloses, to reduce the cellulose crystallinity, and to increase the porosity of the cellulose [2]. The yield of cellulose hydrolysis into glucose by the use of acids or enzymes is generally less than 20% when pretreatment is not carried out, whereas the yield after pretreatment often exceeds 90% [3,4]. In order to increase the yields of sugars and reduce the ethanol production cost, efficient hydrolysis and valorization of hemicellulosic sugars have become important [5]. Once the sugars are under their monomeric form, they can be fermented or chemically converted into molecules of interest such as alcohols (ethanol, butanol, xylitol, arabinol), aliphatic carboxylic acids (succinic acid, lactic acid, levulinic acid), or other molecules [6,7]. After the pretreatment leading to the fractionation of the lignocellulosic biomass and before the conversion into molecules of interest, the sugars undergo a purification step. Among the different processes in lignocellulosic biorefineries, the pretreatment and the purification steps usually represent the most expensive stages and high technical challenges [4,7]. In the literature about lignocellulosic ethanol biorefineries, the notion of pretreatment of the lignocellulosic biomass comes out of the main treatment: the cellulose conversion to ethanol via enzymatic saccharification then fermentation. In this review, instead of pretreatment, the notion of fractionation was favored since the three components of the lignocellulose are of interest.

No cost effective industrial lignocellulosic fractionation process has emerged; they all present some drawbacks such as the formation of fermentation inhibitors, high use of energy or chemicals, waste production, or expensive equipment. The major fractionation processes studied are biological, physical, and chemical, sometimes with some combinations [5]. Among the chemical treatment—hot water, stream explosion, acid, alkaline, organosolv, supercritical fluids, and ionic liquids—mineral acid fractionation is still the method of choice in several model processes [2,5,8,9]. In this review, only mineral acid fractionation was discussed since other acid-like treatments—organic acids, hydrothermal water, supercritical fluids, or acidic ionic liquids—are less or not developed industrially, mainly due to higher cost [10,11,12]. For instance, a newly developed process using an acidic ionic liquid showed promising results for lignocellulose fractionation; however, economic assessments are still to be investigated [13]. These acid-like pretreatments can also produce different molecules from those produced with mineral acid fractionation, which implies different strategies of purification and outlets in other fields of application. For instance, fractionation with organic acids, considered as part of the organosolv processes, such as theprocess developed by the French company CIMV using formic and acetic acids [14], produces sulfur-free and ash-free lignin [15], but modifies the lignin by adding acetyl groups via esterification [16].

The first part of this review deals with the fractionation of lignocellulosic biomass with the use of mineral acids. In the second part, the purification of the resulting lignocellulosic hydrolysates is exposed. A focus was put on the conditions used for the different fractionation and purification processes and the associated yields and purities of the extracted and purified molecules. The cost and environmental impact of the different processes are not detailed in this review; only general trends are provided.

## 2. Mineral Acid Fractionation

### 2.1. Effect and Mechanism

Acid media usually act on lignocellulose by breaking glycosidic bonds and solubilizing hemicelluloses under mild conditions [17] and both hemicelluloses and cellulose under severe conditions [18]. The breakdown of biomass during the pretreatment step or fractionation step, under acidic conditions, facilitates downstream enzymatic hydrolysis by disrupting cell wall structures, driving some lignin into solution, and reducing cellulose crystallinity and chain length [19]. Polysaccharides are sequentially dissolved, then converted into monomeric sugars, and finally, the sugar monomers can be degraded into hydroxymethylfurfural (HMF) for C6 sugars and furfural for C5 sugars depending on the conditions of the reaction [20].

Acid fractionation is especially suitable for biomass with low lignin content [21], as most of the lignin remains in the solid residue. A small part of the lignin is solubilized (about 5–10% of the total lignin) and qualified as acid soluble lignin [22,23]. Depending on the severity of the acidic conditions employed, ester and ether bounds can be broken in the lignin and between the lignin and hemicelluloses [24], but acid media also lead to the precipitation of the lignin and phenolic monomers [25]. When annual biomasses undergo acid treatment, phenol monomers commonly formed are p-coumaric acid and ferulic acid; as for wood biomasses, 4-hydroxybenzoic acid, 4-hydroxybenzaldehyde, vanillin, dihydroconiferyl alcohol, coniferyl aldehyde, syringaldehyde, and syringic acid are the most commonly produced [26].

The only exception regarding the effects described so far of mineral acid catalyst is sulfurous acid, which solubilizes lignin and hemicelluloses, without impacting cellulose [27,28]. It is used in the sulfite process to produce wood pulp composed of highly purified cellulose [29].

### 2.2. Nature of the Acid

Sulfuric acid is the most preferred acid catalyst based on its price, corrosivity, toxicity, and efficiency, but other acids were also studied such as hydrochloric acid, nitric acid, or phosphoric acid [2,30]. The addition of sulfuric acid has been initially applied to remove hemicelluloses either in combination with breakdown of cellulose to glucose or beforeacid hydrolysis of cellulose since the middle of the 20th Century [31]. When acid hydrolysis was performed on sugarcane bagasse, under the same conditions, higher yields of monomeric sugars were obtained with sulfuric acid compared to hydrochloric, nitric, or phosphoric acids [32,33,34,35,36,37].

Superconcentrated hydrochloric acid (15–16 N) can lead to the complete hydrolysis of cellulose within 10 min at 50 °C [38]. However, the lower efficiency of hydrochloric acid [32,33,37], its environmental impact, corrosive properties, and price compared to sulfuric acid strongly limit its application [2].

Nitric acid has not been widely studied. It reduces containment costs relative to sulfuric acid, but its higher cost counterbalances this benefit [31]. Under optimized conditions, nitric acid was less efficient than sulfuric acid at converting hemicelluloses into monomeric sugars [32,34].

Not many data can be found on the use of phosphoric acid for the fractionation of lignocellulosic biomass, though phosphoric acid is promising [35,39,40]. On hyacinth biomass, a study showed that phosphoric acid was more efficient at producing monomeric sugars under the same optimized conditions (concentration, duration, temperature) than sulfuric acid with a yield of 79.9% of monomeric sugars against 75.9% with sulfuric acid [40]. However, on sugarcane bagasse, the reverse result was obtained with sulfuric acid, leading to the production of more monomeric sugars than with phosphoric acid with even milder conditions [32,35] Neutralization of phosphoric acid hydrolysates with sodium hydroxide leads to the formation of sodium phosphate. This salt can remain in the hydrolysates because it is used as a nutrient by microorganisms during the following step of monosaccharide fermentation. This has two advantages: no filtration step to remove the precipitated salt is required, and it decreases the addition of nutrients to run the fermentation step [2].

### 2.3. Conditions and Yields

An optimal balance in the hydrolysis conditions has to be found to maximize the hydrolysis of polysaccharides to obtain monomeric sugars and prevent further degradation of these monomeric sugars into degradation products [37,41]. For the conditions of fractionation, four main parameters vary: the concentration of the acid, the solid:liquid ratio (S:L ratio), the temperature, and the reaction duration. Other parameters, such as the biomass particle size [36] or the agitation, are also important parameters influencing the efficiency of the fractionation, but they are less subject to variation in the different studies found in the literature.

Acid treatment is carried out in the presence of high and low concentrations of acid and at high and low temperatures [42]. Low concentrations of acid are often associated with high temperatures, whereas high concentrations of acid are carried out at low temperatures with a higher S:L ratio.

#### 2.3.1. Low Concentrations of Acid and High Temperatures

Dilute acid fractionation processes usually use an acid concentration range of 0.5 to 8% (*w*/*w*), a S:L ratio range of 1:5 to 1:20 (*w*/*v*), a temperature range of 100 to 200 °C, and a reaction duration range of 5 to 300 min.

**Figure 1:**
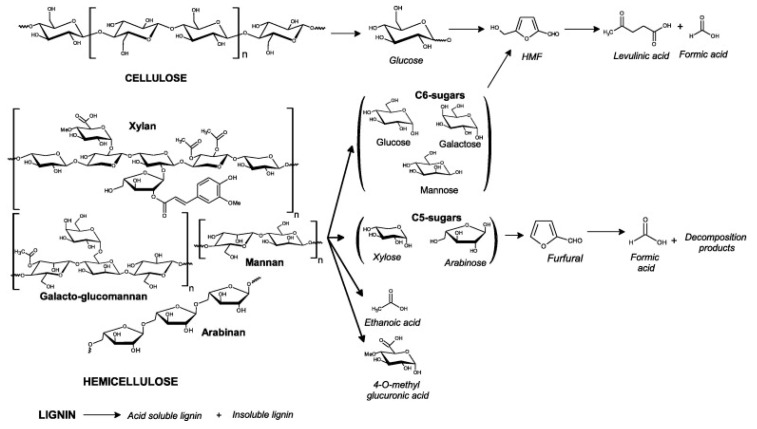
**Formation of** degradation products from lignocellulosic biomass under dilute acid pretreatment at high temperatures [44]. HMF, hydroxymethylfurfural.

Dilute sulfuric acid fractionation generally leads to the solubilization of the hemicelluloses and a small fraction of the lignin, the hydrolysis of the solubilized hemicelluloses, and the decrease in cellulose crystallinity [1,22]. Therefore, dilute acid fractionation eliminates or reduces the need for hemicellulase enzyme mixtures for hemicellulose saccharification [22]. In parallel, during dilute acid fractionation, the majority of lignin remains as a solid residue, and only some ether and ester linkages are cleaved, generating low molecular-weight lignin fragments with increased hydroxyl group content [43]. When dilute acid hydrolysis is run at high temperatures or for a long period of time, it can break down cellulose as well [30,44]. During the acid hydrolysis of lignocellulosic biomass at a given acid concentration, S:L ratio, temperature, and the reaction duration monitoring is important. Indeed, under acidic conditions, cellulose is first hydrolysed into glucose, which in turn can be converted into HMF, and HMF can finally be degraded into levulinic acid and formic acid (Figure 1). In parallel, hemicelluloses are also hydrolysed into monomeric sugars (xylose, arabinose, galactose, mannose, glucose, etc.), and acetate groups attached to the hemicelluloses are released, as well as uronic groups producing acetic acid, glucuronic acid, and galacturonic acid [45]. Monomeric C5 sugars are then converted into furfural, which in turn can be converted into formic acid and other degradation products [44,46]. There is an optimum duration after which the hydrolysis of more hemicelluloses and celluloses into monomeric sugars does not compensate the loss of monomeric sugars being converted into furan degradation products [22]. The variation of one of the three parameters—acid concentration, S:L ratio, or temperature—affects the optimum duration of the hydrolysis. For instance, at 1% (*w*/*w*) sulfuric acid, the higher the temperature, the faster the maximum yield of glucose was reached, from 50 min at 170 °C to 2 min at 220 °C [41]. Longer reaction durations led to a decrease inthe concentration of glucose due to its degradation [41]. Similarly, with all other hydrolysis variables constant, an increase in the acid concentration led to a faster rate for the hydrolysis of the hemicelluloses [37].

Inspired by the pulp and paper industry, a combined severity factor (CSF) was developed by Chum et al. (1990) for dilute acid treatment taking into account temperature, acid concentration, and duration of the reaction as detailed in Equation (1) [47]. On corn stover, a CSF in the range of 1.4–1.8 was optimal for the xylose yield in the acid hydrolysate, and lower or higher CSF reduced this yield due to incomplete solubilization and hydrolysis or due to the degradation of monomeric sugars , respectively [48].

(1) $$\mathrm{CSF}=\log\left\{t\;.\;\exp\left(\frac{T_{H}-T_{\mathrm{Ref}}}{14.75}\right)\right\}-\mathrm{pH}$$

where t is the reaction duration in min, $T_{H}$ is the reaction temperature in °C, $T_{\mathrm{Ref}}$ is the reference temperature, most often 100 °C, and pH is the initial pH value (calculated from the mineral acid concentration).

The efficiency of the fractionation has sometimes been defined by the sum of the monomeric sugar concentrations (glucose, xylose, arabinose) divided by the sum of the fermentation inhibitor concentrations (furfural, HMF, acetic acid) [34,35,49]. It is interesting to anticipate the yield of the following step of sugar fermentation based on this ratio and in this way to compare the efficiency of the fractionation conditions. However, different inhibitors have different inhibition thresholds depending on the fermentation enzymes or microorganisms used; for instance, furans usually decrease their activity at lower concentrations than acetic acid [46,50]. Besides, the final concentration of monomeric sugars is highly dependent on the S:L ratio used for the dilute acid hydrolysis.

Studies also presented the yield of monomeric sugars after dilute acid fractionation and enzymatic saccharification or the yield of ethanol after fermentation of the sugars [51,52]. Comparison between the different fractionation processes is valid only if the same enzymatic saccharification or fermentation conditions are used, but as there is no standard process, this is rarely the case. Besides, some studies focused on the production of other molecules than ethanol from the enzymatic conversion of monomeric sugars such as hydrogen [49] or xylitol [53].

For these reasons, to compare the different acid fractionation processes, the yields of monomers obtained after the dilute acid hydrolysis (the production monomeric sugars over their total potential regarding the polysaccharides content in the initial biomass) appeared to be of interest. As purification is often required between the saccharification step and the valorization of the monomeric sugars, it would be valuable to look for the highest possible yields for the monomeric sugars after the fractionation step and not consider their purities or concentrations. The results of some studies are displayed in Table 1, with a focus on sugarcane bagasse (SCB) for lignocellulosic biomass and sulfuric acid for the acid used, for an easier comparison between the hydrolysis conditions. SCB is one of the most studied lignocellulosic biomass in the literature. It contains mainly three sugars—glucose, xylose, and arabinose—and its hemicelluloses contains a very low amount of glucose [47], so the quantified amount of glucose in the acid extract can be correlated to the hydrolysis of cellulose. Other biomasses are presented for comparison. When the results of the hydrolysis were expressed in g/L in the literature, they were converted by calculation to yield of monomeric sugar following Equations (2) and (3):

(2) $$Y_{\mathrm{glucan}}=\frac{\left[\mathrm{glucose}\right]}{C\;1.11\;r_{S:L}}$$

with [glucose], the concentration of glucose in the acid hydrolysate (g/L), C the cellulose content in the initial biomass (% dry solid(DS)), and r_S:L_ the solid:liquid ratio of the hydrolysis (g/L). C has to be corrected by a factor of 1.11 to represent the initial potential in glucose of the biomass as a molecule of water is added to glucose during the hydrolysis of cellulose.

(3) $$Y_{\mathrm{hemicelluloses}}=\frac{\left[\mathrm{xylose}\right]+\left[\mathrm{arabinose}\right]}{H\;1.14\;r_{S:L}}$$

with [xylose] and [arabinose] the concentrations of xylose and arabinose in the acid hydrolysate (g/L), H the hemicelluloses (xylan and arabinan) content in the initial biomass (%DS), and r_S:L_ the solid:liquid ratio of the hydrolysis (g/L). H has to be corrected by a factor of 1.14 to represent the initial potential in xylose and arabinose of the biomass, respectively, as a molecule of water is added to xylose and arabinose during the hydrolysis of hemicelluloses.

Sulfuric acid was found to be the most efficient to yield monomeric sugars among all the acids tested (Table 1). The lignocellulosic biomass treated had a significant impact on the yield of monomeric sugars (Table 1). For instance, with the same fractionation conditions, glucose yield reached 33% for bermudagrass, whereas it was 10% for rye straw [52].

Dilute acid treatments are considered inexpensive given the low cost of acids, are relatively efficient given the hydrolysis of hemicelluloses into monomeric sugars (generally yield of about 90%), and given the yield of the following step: the enzymatic hydrolysis of cellulose. They are also fast compared to other fractionation processes, for instance mild alkaline fractionation that usually requires several hours of reaction duration [54,55,56]. However, they are carried out at a low S:L ratio and at high temperatures, which impact the cost efficiency of the process. Besides, acids under high temperatures are corrosive, so resistant reaction vessels are required. This process also generates fermentation inhibitors (furans, aliphatic carboxylic acids, phenol derivatives) that have to be removed before the enzymatic reaction step. Purifying these molecules for further valorization can also enhance the profitability of the acid fractionation process.

The dilute acid fractionation process was also used to produce the degradation products of sugars, which present a high value, some being referred as platform chemicals by the U.S. Department of Energy, such as levulinic acid or furfural [6]. The Biofine process, developed by Fitzpatrick in the 1990s, produced levulinic acid and furfural from a dilute acid treatment of lignocellulosic biomass without enzymatic hydrolysis or fermentation step [57,58]. Sulfuric acid at concentrations between 1 and 5% (*w*/*w*) was contacted with biomass for short periods of time (from a few seconds to a few minutes) in a two reactor system at high temperatures (in the range of 195–230 °C) to obtain high yields of levulinic acid and furfural from the degradation of the hexoses and pentoses [57,58]. It led to the conversion of approximately 50% of the mass of six-carbon sugars to levulinic acid, with 20% being converted into formic acid and 30% being incorporated in the residual “char” material, which also contains all of the acid insoluble lignin and 50% of the pentoses that did not convert into furfural [4]. A commercial facility run by GF Biochemicals in Caserta, Italy, processes 50 tons per day of waste paper, municipal wastes, and agricultural residues to produce levulinic acid and furfural based on the Biofine process [4].

#### 2.3.2. High Concentrations of Acid and Low Temperatures

The concentrated acid hydrolysis process appeared to be an interesting process for saccharification of lignocellulosic biomass, as it leads to high yields of monomeric sugars, low levels of production of fermentation inhibitors, and good flexibility regarding the different raw material treated compared to the dilute acid fractionation [18]. Low temperatures (typically under 60 °C) and high S:L ratios (from 1:2.5 to 1:10 (*w*/*v*)) are generally used, improving significantly the cost effectiveness of the pretreatment [21,42,61]. No enzymatic saccharification is required to reach the same yields of monomeric sugars as with dilute acid hydrolysis. For instance, rice hulls treated with concentrated H_2_SO_4_ 67% (*w*/*w*) at 25 °C for 3 h gave higher yields of monomeric sugars (62%) than dilute acid treatment (H_2_SO_4_ 1% (*v*/*v*), 121 °C, 15 min) followed by enzymatic saccharification (60% yield) [51]. Concentrated mineral acids such as sulfuric acid and hydrochloric acid are widely used for treating lignocellulosic materials because they are powerful agents for both hemicellulose and cellulose hydrolysis. The major drawback of these concentrated acids is their corrosive nature and the need to recycle acids to lower the cost of fractionation [21,42]. Special acid resistant materials for the vessels are still to be investigated, ceramic or carbon-brick lining having great potential, for example [8].

As for dilute acid treatment, the four main parameters (acid concentration, S:L ratio, temperature, and hydrolysis duration) have to be selected carefully in order to maximize the yield of solubilization and hydrolysis of the polysaccharides. For instance, from corn stover treated under H_2_SO_4_ 70% (*w*/*w*) at 50 °C for 20 min, 90% yield for the monomeric sugars was achieved with an S:L ratio of 1:50 (*w*/*v*), but when the S:L ratio was increased to 1:10, then the yield of monomeric sugars dropped to 65% [20]. With the same sulfuric acid concentration, an intermediate S:L ratio of 1:20 (*w*/*v*) and an increased temperature to 70 °C, total conversion into monomeric sugars was achieved [20]. Using a Taguchi experimental design, Liu et al. (2012) determined that the concentration of sulfuric acid followed by the S:L ratio were the most influential parameters for the recovery of glucose and xylose [61]. It was also shown that the production of sugar degradation products (furans and aliphatic carboxylic acids) was more influenced by the temperature.

In order to be competitive with fossil fuels, the main challenge for the use at industrial scale of the concentrated acid fractionation of lignocellulosic biomass to produce ethanol is the recycling of the acid, which has risen from 80 to 97% in the process in the last 50 years, but the cost of the recycling process remains high [4,18]. In theory, sulfuric acid acts during lignocellulosic biomass hydrolysis as a catalyst, so in principle, no sulfuric acid should be consumed during this process [62]. However, some acid still has to be reintegrated in the process in addition to the recycled acid due to unavoidable losses (for instance, through absorption by the biomass or salification of inorganic cations) [62].

### 2.4. Industrial Applications

Dilute sulfuric acid fractionation under fairly mild conditions received the main focus from manufacturers and research institutes. For instance, the National Renewable Energy Laboratory (NREL), from the U.S. department of Energy, established an exhaustive public report with the technical and economic feasibility of such fractionation [19]. The process described uses co-current dilute acid fractionation of lignocellulosic biomass (corn stover) to liberate the hemicelluloses, followed by enzymatic hydrolysis (saccharification) of the remaining cellulose, followed by fermentation of the resulting glucose and xylose to ethanol. Overall, the total acid loading was 22.1 mg/g dry biomass, and the effective sulfuric acid concentration in the fractionation reactor was estimated at 0.3–0.4% *w*/*v*, which may allow for the use of lower cost metallurgies in the reaction zone. All acetate groups bound to the hemicelluloses were released under the form of acetic acid; 5% of the xylose was converted to furfural; and 5% of the lignin was solubilized (Table 2).

Industrial development of the concentrated acid fractionation is at its beginnings. Some companies such as Biosulfurol Energy attempted to commercialize the concentrated acid process for the fractionation of lignocellulosic biomass in order to produce ethanol [63], but eventually failed (the company was dissolved in 2016). The market appears to be led by three companies: BlueFire Renewables, Virdia, and Renmatix [64]. The companies claim to be economically competitive with the production of sugars from traditional agricultural sources such as sugarcane. For instance, the process from Virdia claims to produce monomeric sugars at 0.25 $/kg from lignocellulosic wastes compared to 0.45 $/kg from sugarcane [64].

Arkenol gave detailed conditions in two patents regarding the fractionation with concentrated acid followed by dilute acid hydrolysis. Sulfuric acid 70–77% (*w*/*w*) should be added to the biomass in order to achieve a ratio of acid to cellulose and hemicelluloses of at least 1.25:1 (*w*/*w*) [65,66]. After the decrystallization or solubilization stage, if the concentrated acid fractionation did not lead to a 100% yields of monomeric sugars, the concentrated acid can be diluted, to a concentration of about 20–30% for a second hydrolysis to completely convert the solubilized cellulose and hemicelluloses into monomeric sugars [66]. BlueFire Renewable’s process is based on the Arkenol patent [66]; their plant, in operation since 2002 in Izumi, Japan, was already producing over 80,000 L of ethanol 95% in 2004 (https://bfreinc.com/production-plant/, accessed on 25 November 2017). Another patent gave similar hydrolysis conditions and provided detailed solubilization yields of polymeric sugars and conversion yields into their monomeric forms depending on the conditions (acid concentration, S:L ratio, temperature, and experiment duration) used during the concentrated acid hydrolysis, then the dilute acid hydrolysis [20]. Similar processes have been used in other studies; for instance, Sun et al. (2011) produced an acid hydrolysate from bamboo with successive concentrated acid, then dilute acid hydrolysis and obtained a monomeric sugar yield of 81.6% [67].

The combination of concentrated acid hydrolysis and diluted acid hydrolysis for the saccharification of cellulose and hemicelluloses has been optimized and proven to be so efficient that it became a standard method for the characterization of lignocellulosic biomass carbohydrates and lignin, developed by the National Renewable Energy Laboratory, Colorado, USA [23]. A first hydrolysis with concentrated sulfuric acid (72% *w*/*w*) and an S:L ratio of 1:10 (*w*/*v*) at 30 °C for 1 h, followed by a dilution to reach 4% (*w*/*w*) sulfuric acid to run a second hydrolysis at 121 °C for 1 h, led to a complete saccharification of the cellulose and hemicelluloses. Incomplete saccharification, detected by the presence of cellobiose, can be corrected by a longer diluted acid hydrolysis, while over time, hydrolysis can be detected by the follow-up of furfural and HMF [23].

## 3. Purification Routes Applied to Lignocellulosic Acid Hydrolysates

Monomeric sugars are the target molecules to valorize from lignocellulosic acid hydrolysates. Depending on the concentration of the mineral acid, it can be of interest to recycle it. The lignocellulosic acid hydrolysates are also constituted of other molecules, which are fermentation inhibitors, such as acetic acid, decomposition products from monosaccharides (furans and aliphatic carboxylic acids), and phenolic compounds.

Purification requirements (purity, yield, and cost) have to be considered regarding the final objective of the process. For some applications, the removal of fermentation inhibitors before fermentation is not necessary. For instance, the purification of a hemicellulosic acid extract by several methods (overliming, adsorption on charcoal or on resins) did not lead to higher xylitol yields by fermentation, compared to a simple pH adjustment to 5.5 before fermentation [68]. For other fermentation reactions, for instance glucose or xylose to ethanol, concentrations of furans greater than 1 g/L strongly inhibits the enzymes [69], so lower concentration for these molecules are targeted after the purification step. Since the detoxification process can be expensive and take a large portion of the whole ethanol production cost, the selection of the detoxification method is of major importance [1].

The purification cost can be compensated by the value of the molecules to remove. Their valorization would contribute to the global efficiency of the lignocellulosic biorefinery.

### 3.1. Alkalinization/Overliming

Alkali treatment is suitable for dilute acid hydrolysates because of the high quantity of base required to adjust the pH. It leads to great improvement in fermentability of the hydrolysates by chemically converting fermentation inhibitors [64] or precipitating and removing them by centrifugation or filtration [3,70,71]. Sodium hydroxide, potassium hydroxide, calcium hydroxide, and ammonia have already been tested for neutralization or alkalinization of lignocellulosic acid hydrolysate [64].

The best conditions to use sodium hydroxide are high temperatures in combination with moderate pHs or moderate temperatures in combination with high pHs. For instance, a pH adjustment to nine at 80 °C were the best conditions, with an increase of 110% in ethanol production from a lignocellulosic dilute acid hydrolysate compared to the value of the reference [70]. However, under similar conditions, sodium hydroxide treatment has so far been less efficient than overliming [70].

Overliming, i.e., the addition of calcium hydroxide, is considered tobe one of the most efficient processes for the removal of fermentation inhibitors [1,70]. In the overliming process, the hydrolysate is detoxified by the addition of calcium hydroxide to adjust the pH to 9–10, leading to the precipitation of some of the furans and phenolic compounds, which are recovered by centrifugation or filtration [68,72]. The resulting hydrolysate is readjusted to pH 5.5 with dilute sulfuric acid in order to carry out fermentation. An overliming treatment (pH adjustment to 10.5, 90 °C, 30 min) on an acid and enzyme treated rice hull hydrolysate, not only increased the maximum yield of ethanol, but also reduced the duration required for the maximum production of ethanol in the case of a simultaneous saccharification and fermentation process [51]. However, a study showed that overliming led to a drastic reduction of potential fermentable sugars, both at pH 5.5 or 10, with 28 and 47% sugar elimination, respectively [73]. This degradation of fermentable sugars resulting in moderate yields of ethanol cannot be acceptable for industrial implementation, as well as the formation of large amounts of gypsum [1,70,73]. In the NREL technical report mentioned in the previous section, it was reported that a significant amount of sugars (as much as 13%) could be lost because of side reactions occurring at high pH or it is pressed out with the wet gypsum [19].

Alkalinization with ammonia is an alternative to overliming. It presents the advantage of using milder conditions (pH 9 and 55 °C) and reducing the amount of precipitate formed [70]. Besides, detoxification with ammonia was found to be more efficient than with sodium hydroxide and calcium hydroxide regarding the yield and productivity of ethanol [64,65]. Detoxification with ammonia was taken as a reference by NREL in their technical report (2011) [19]. In this process, ammonia was added to the lignocellulosic acid hydrolysate to raise its pH from about one to five before enzymatic hydrolysis. No precipitation occurred, and fermentation studies have indicated that there was no benefit to over-conditioning at high pH when using ammonia, so the hydrolysate was simply adjusted to the pH of the enzymatic hydrolysis in one step. Ammonia is more expensive than lime, but they claimed that the economic benefits of reduced sugar loss and reduced capital cost make ammonia the most economical alternative. It can also possibly reduce nitrogen requirements during the fermentation step, but this has not been demonstrated yet.

Despite the optimization realized, alkalinization processes present some drawbacks including the large amount of base required, the impossibility to recycle the acid catalyst for the hydrolysis step, their lower efficiency compared to other purification techniques, and the acetic acid remaining fully in the hydrolysates [71,72]. Nevertheless, most industrial purification methods of lignocellulosic acid hydrolysates begin with a partial or complete neutralization of the inorganic acid, then other methods such as ion-exchange, adsorption, chromatography, or crystallization are used to purify the sugars [71].

### 3.2. Evaporation

Evaporation is a simple procedure to remove acetic acid, furfural, and other volatile components from the hydrolysates, but phenolic monomers and lignin degradation products cannot be removed [74]. It can decrease the concentrations of acetic acid and furfural below their inhibitory levels for some applications such as the fermentation of xylose to xylitol. For instance, the concentration of *Eucalyptus* wood dilute acid hydrolysate to five-fold by evaporation at 70 °C led to the removal of 97.7% of furfural, 61.3% of acetic acid, and 22.8% of HMF [75]. Evaporation under acidic conditions (pH 1) favored the evaporation of acetic acid, which is volatile only under its protonated form, but on the other hand, acidic conditions were less favorable for HMF removal [73].

### 3.3. Liquid/Liquid Extraction

Several organic solvents (e.g., chloroform, n-hexane, and ethyl acetate) have been tested for the removal of fermentation inhibitory compounds from lignocellulosic acid hydrolysates using various hydrolysate:solvent ratios (2:1, 1:1, 1:2, 1:3, (*v*/*v*)) [73]. Increasing the hydrolysate:solvent ratio improved the efficiency of the removal of the fermentation inhibitors until 1:2 (*v*/*v*), and a further increase did not produce noticeable changes. Overall, ethyl acetate was the most efficient solvent with removal rates of 50% for phenolic compounds, 94% for furfural, and 40% for HMF. The sugar loss (8%) was less important than with alkalinization/overliming or evaporation processes.

In another study, four extractions with a lignocellulosic acid hydrolysate:ethyl acetate ratio of 1:1 (*v*/*v*) were efficient to remove all the phenolic monomers and the furfural, and also led to the same removal level of acetic acid as evaporation [74]. Based on the yield of ethanol in the following fermentation stage, liquid:liquid extraction was far more efficient (3-fold factor) than evaporation. However, the level of acetic acid left in the solvent extracted acid hydrolysate was still too high in this study, as it led to a longer fermentation duration than with an acid hydrolysate free of acetic acid [74]. Besides, the high consumption of solvent and the necessity to recycle it are the main limiting factors for the economic efficiency of the liquid/liquid extraction process.

### 3.4. Adsorption

Purification through adsorption processes is based on the difference of affinity among the various molecules from a mixture with a sorbent. Two sorbents were mainly studied to separate lignocellulosic acid extract components: activated charcoal (AC) and resins. The aim of using these sorbents on lignocellulosic acid extracts is to adsorb sugar fermentation inhibitors such as phenolic compounds, furans, and to a lower extent, acetic acid.

#### 3.4.1. Activated Charcoal

The most important parameter for an efficient adsorption with AC is the hydrolysate:AC ratio [76]. Optimum ratios to ensure good removal rates of impurities with no adsorption of sugars appeared to be about 200:1 to 50:1 (*w*/*w*) depending on the concentration of the hydrolysate components [50,72,77]. pH also strongly influences the adsorption process, weak organic acids (phenols, acetic acid) being most readily adsorbed in the non-ionized state and consequently low pHs favor their adsorption, whereas the ionized form of the weak acids are poorly adsorbed at high pHs [50,73,78]. Contact time between activated charcoal and hydrolysates for an optimal adsorption was at least 20 min, and equilibrium was reached in about 60 min [76]. The temperature also influences the adsorption of the inhibitors, such as aromatic compounds. For instance, temperatures close to room temperature led to better adsorption of phenolic compounds by about 20% than an adsorption experiment run at 60 °C on an acid *Eucalyptus* wood hydrolysate [77].

The addition of AC to neutralized *Eucalyptus* wood acid hydrolysates with a hydrolysate:AC ratio of 200:1 (*w*/*w*) at 40 °C led to about 80% lignin adsorption, whereas xylose was almost totally recovered in the hydrolysate (less than 2% adsorption) [77]. The adsorption of lignin improved by 28% the consumption of xylose during the downstream fermentation step [77]. When the production of ethanol is the target of the biorefinery, the lignocellulosic acid hydrolysates are often pH adjusted to 5.5 before AC adsorption as the fermentation of the sugars occurred at this pH [68,72]. This rise in pH of the hydrolysates can also be beneficial for the adsorption of the inhibitory compounds, as low pH can lead to the adsorption of sulfuric acid, which makes the surface of the AC less hydrophobic, thus reducing the adsorption of the inhibitory compounds [79].

The comparison of alkalinization, overliming, evaporation, liquid/liquid extraction, and adsorption with AC showed that adsorption with AC was the most efficient method for the removal of fermentation inhibitors while minimizing sugar losses [73]. The use of adsorption also decreases the cost of the process as low temperatures (20 to 40 °C) are used, as well as low ratios of hydrolysate:charcoal (200:1 to 50:1 (*w*/*w*)).

On a synthetic solution of 20% (*w*/*w*) sulfuric acid, containing glucose, furfural, HMF, and acetic acid, AC was found to adsorb furans and acetic acid more effectively than a cation exchange resin [79]. However, in order to give value to the adsorbed fermentation inhibitory compounds, desorption of these compounds is important as well. Usually, in the adsorption/desorption process, regeneration or desorption time is comparable to the duration of the loading or adsorption step. Unlike cation exchange resin, the regeneration of granulated activated charcoal (GAC) with water was not feasible; ethanol 50% (*v*/*v*) was required to desorb furans and acetic acid in a limited duration [79]. Overall, AC led to the best performance compared to resins when high purity is required, and when ethanol can be used to regenerate the adsorbent.

#### 3.4.2. Resin

Adsorption by resins can also be used to remove acetic acid, phenolic, and furanic compounds from lignocellulosic acid extracts. The mechanisms involve hydrophobic interactions due to the resin matrix, as well as ionic bonds for anion- or cation-exchange resins. Studies from the literature usually assessed the efficiency of the adsorption on the resins based on the removal rates of the fermentation inhibitors and on the yield of ethanol after the downstream fermentation step. It is also of interest to follow the desorption of the inhibitors, in order to produce a fraction with high added value molecules that can be further valorized.

When the pH was adjusted to 5.5 before the adsorption step, anion-exchange resins presented the best removal of fermentation inhibitors and the best ethanol yields during the downstreamfermentation step compared to overliming or adsorption on activated charcoal, nonionic resins, or cation-exchange resins [68,72,80]. A higher pH, e.g., pH 10, before the adsorption on anion-exchange resin, increased the adsorption of aliphatic carboxylic acids and phenol by making them negatively charged; it also increased the adsorption of furan derivatives [80]. The same study showed that increasing the pH before the adsorption was also interesting to minimize the sugar loss by adsorption (no loss at pH 10), as ionized aliphatic carboxylic acids, phenols, and inorganic ions such as sulfate efficiently competed for the positive sites in the anion-exchange resin. The hydrolysate:resin ratio also had a substantial impact on the adsorption of fermentation inhibitors and the yield of ethanol afterfermentation. The removal rate reached 96% for aliphatic carboxylic acids, 68% for furfural, 65% for HMF, and 81% for phenolic compounds with a hydrolysate:anion-exchange resin ratio of 25:8 (*v*/*w*).

In order to reduce the use of chemicals, the adsorption can be run without neutralization of the lignocellulosic acid extract. Without neutralization, anion-exchange resins presented less interest because such resins adsorbed preferably sulfate ions instead of anions of weak organic acids (aliphatic carboxylic acids or phenolate ions), and thus, low removal rates were obtained [79]. On cation-exchange and nonionic resins, acid hydrolysates without pH adjustment were more favorable for sugar purification as sugars were very weakly adsorbed, whereas the adsorption of furfural and HMF was more favorable [69,80,81]. The adsorption of phenolic compounds was also high given that these molecules were uncharged at low pHs [69,80,81]. However, with this process, acetic acid was not separated from the sugars [79,80]. Furfural loading capacity was about 2-fold higher on hydrophobic adsorbent, for instance nonionic resin made of polystyrene-divinylbenzene (PS-DVB), than on more hydrophilic adsorbent such as methacrylic ester resin, suggesting that the predominant mechanism of attraction between the resin and the furfural is hydrophobic attraction [69]. Besides, the adsorption of acetic acid, furfural, and HMF on cation-exchange or nonionic resin is higher in 20% (*w*/*w*) sulfuric acid than in water [79]. This is due to the “salting out effect” corresponding to an increase in the adsorption of hydrolysate components with increasing ionic strength in the liquid phase [79]. Indeed, in an aqueous solution, water molecules preferably solvate sulfuric acid molecules instead of the neutral molecules that are “salted out” (i.e., adsorbed) onto the resin [79].

Using a synthetic hydrolysate, containing 20% (*w*/*w*) sulfuric acid, the loading capacity of a cation resin was three-times less than the capacity of a nonionic resin and 14-times less than the capacity of AC [79]. However, a sorbent has to be selected not only based on its ability to adsorb the fermentation inhibitors from a lignocellulosic acid hydrolysate, but also based on its ability to desorb them with the appropriate solvent. The increase in concentration of a desorbed molecule in the eluent during the desorption step compared to its concentration during the loading step in the feed, called overshoot, is due to lower eluent volume required to desorb the adsorbed molecules than the volume of the solution fed on the column during the adsorption. This phenomenon is particularly interesting as it decreases the necessity to further concentrate the molecules before the downstream purification and valorization steps. The regeneration of a strong cation-exchange resin can be carried out with water alone, with a comparable duration to the loading step, and with a small overshoot, making the use of strong cation-exchange resin favorable [79]. Water can desorb the adsorbed fermentation inhibitors due to the salting out phenomena vanishing as the ionic strength of the eluent is reduced.

Contrary to cation-exchange resin, regeneration of nonionic resin andGAC with water is not feasible; an organic solvent is required, involving a higher cost for this process. All organic solvents were not suitable for the desorption of fermentation inhibitors from nonionic resin and GAC; some like n-propanol and n-butanol can be possibly significantly adsorbed on the hydrophobic adsorbent [69]. Batch desorption with an ethanol:nonionic resin ratio of 15:1 at 50 °C with stirring for 90 min led to 95% furfural desorption [69]. Regeneration of nonionic resin in a column performed with 75% acetone at room temperature desorbed 85% of the acid soluble lignin [81]. Another study showed that 50% ethanol was efficient to desorb furans from a nonionic resin or GAC in a column and led to an overshoot of furans by a factor of three during the desorption [79].

### 3.5. Low Pressure Chromatography

Several eluents and a process set-up with numerous steps are used for adsorption: feed loading, rinsing, desorption, regeneration, and equilibration; chromatography requires only one eluent and an easier process set-up—feed loading and elution—which generally leads to lower economic and environmental cost [82]. If two types of particles differ in their adsorption rate, separation of them may be accomplished by selectively adsorbing one species on the sorbent. The reversibility of adsorption allows chromatographic separation of particles contained in a solution [83,84].

After acid fractionation on lignocellulosic biomass, the chromatographic resin process can be used to separate acid and sugars on a first step as demonstrated by the Arkenol patents for example [20,66]. Gel type PS-DVB strong acid cation-exchange resin with H^+^ as counter-ion was used during this step under chromatographic conditions with water as the eluent [65,85]. The water flow rate on the resin bed was about 2 to 5 m/h, and the temperature was kept between 40 and 60 °C. Sugars were slowed down by the resin while sulfuric acid was not retained. On batch elution, depending on the different conditions tested, it was possible to reach 90–93% sugar purity and 90–96% sugar recovery; 95–96% acid purity and 97–99% acid recovery [66]. A plant using this technology is run by BlueFire Renewable in Izumi, Japan, with a capacity of 80,000 L/year of ethanol 99.5% (*v*/*v*) (https://bfreinc.com/, accessed on 25 November 2017). The performance of the chromatographic separation was found to decrease with increasing concentration of sulfuric acid from 20% to 70% (*w*/*w*) [86]. Besides, as explained previously, lignocellulosic biomass concentrated acid extraction is often followed by a more diluted acid hydrolysis in order to optimize the recovery of the monosaccharides. Therefore, lignocellulosic acid hydrolysates containing 20% (*w*/*w*) sulfuric acid were often studied as the feed of the chromatographic step for the monosaccharides/acid separation. A process was developed to recycle the acid after the chromatographic separation and send it back to the beginning of the process for the lignocellulosic biomass hydrolysis, and 92% of the acid needed for initial hydrolysis was obtained through recycling [86]. Moreover, the water removed from the acid can be reused as the eluent for the chromatographic separation, decreasing the water consumption of the whole process by 60% [86].

Some fermentation inhibitors (acetic acid, furans) can also be removed from lignocellulosic acid hydrolysates along with sulfuric acid by the chromatographic process. The use of a cation-exchange resin with a PS-DVB matrix induced the elution of sulfuric acid first, then the monomeric sugars, and finally, acetic acid and furans, whereas the use of an anion-exchange resin with a polyvinylpyrrolidone matrix led to the elution of monomeric sugars first, followed by the fermentation inhibitors, and finally, sulfuric acid [87,88]. After fermentation of the resulting purified sugar mixtures, the ethanol yields were as good as on a synthetic sugar solution and better than the acid hydrolysates purified by overliming [88].

Resins were also studied for the separation of the monomeric sugars constitutive of lignocellulosic acid extracts after acid removal. The adsorption behavior of glucose, xylose, and arabinose has been tested on several strong acid cationic exchanger with various cross linking degree (DVB content of 4, 6, and 8%) and different counter-ions (K^+^, Ca^2+^, Fe^3+^) for the separation of glucose, xylose, and arabinose [89]. Resins with Ca^2+^ as counter-ions were the most suitable for the separation of the monomeric sugars after adsorption isotherm determination tests; 6% DVB resin being more efficient for arabinose/xylose separation and 8% DVB resin being more efficient for xylose/glucose separation [89]. Other adsorption isotherm experiments confirmed the higher potential of Ca^2+^ for strong acid-exchange resin over Na^+^ to separate glucose, xylose, and arabinose [90]. With an Amberlite IRP69-Ca^2+^ packed column and following the eluent volume at which the fractions were determined, recoveries and purities of about 90% for glucose, xylose, and arabinose were obtained by batch column chromatography from both a synthetic solution and a pine branch hydrothermal liquefaction extract [90]. Continuous chromatography via a multi-column system such as a simulated moving bed (SMB) is a classic industrial method to separate sugars and can be completed by crystallization to reach a high level of purity for the different sugars [71,84].

### 3.6. Cross-Flow Membrane Filtration

Membrane filtration was extensively studied in lignocellulosic biorefineries using alkaline fractionation processes, for instance for the purification of spent liquor in a sulfite pulp mill to remove lignosulphonates [91], but this technology was used in a more limited extent to purify lignocellulosic dilute acid hydrolysates. Monosaccharides from acid hydrolysates cannot be purified in one membrane filtration step. Membranes with a molecular weight cut-off (MWCO) of 10–50 kDa can be used to separate lignin or proteins in the retentate from the monomeric sugars, sulfuric acid, and the other impurities (acetic acid, furans) in the permeate [71,92]. Membranes with MWCO of 150–300 Da can retain monomeric sugars (glucose, xylose, arabinose), while acetic acid and furans pass through the membranes [93,94].

Organic flat sheet membranes of 10, 20, and 50 kDa were efficient to totally retain macromolecules (lignin and proteins) from a wheat bran acid extract, unlike ceramic tubular membranes with lower MWCO (8 and 15 kDa), where the retention of macromolecules was not total [71]. In another study, several successive concentrations by a volumic reduction factor of 3.6 of a wheat bran acid extract were run on a 10 kDa organic membrane (polysulfone filtration layer with polypropylene support material), and the flux was on average about 10 L/h/m^2^ showing good reproducibility [92]. Afterward, diafiltration with 2.5 diavolumes was required in order to maximize sugar recovery in the permeate (99% recovery). Filtration of rice straw dilute acid hydrolysate adjusted to pH 3 on an organic spiral wound membrane with an MWCO of 150–300 Da led to total retention of glucose and very high retention of xylose and arabinose (over 94%), while acetic acid and furans totally passed through the membrane [93]. The separation performance decreased when the operating temperature was increased from 25 to 40 °C.

During the filtration of lignocellulosic hydrolysates, fouling occurred on the membrane, changing the initial properties of the membrane (membrane permeability and selectivity), and membrane cleaning was necessary to recover its initial properties. Membrane cleaning with 0.01 N of sodium hydroxide and rinsing with water was enough to recover the initial water flux after the filtration of rice straw dilute acid hydrolysates [93].

### 3.7. Electrodialysis

Electrodialysis (ED) has been applied to the purification of monomeric sugars from lignocellulosic acid hydrolysates, to remove sulfuric acid and acetic acid, but partial or complete neutralization before ED was still required to eliminate macromolecules (lignins and proteins) that could precipitate [62,71].

A study on sugarcane bagasse dilute acid hydrolysate treated by ED led to a sulfuric acid recovery of 88%, and the loss of sugar was less than 5% [62]. Successive ED runs on wheat bran dilute acid hydrolysates showed good reproducibility and led to similar results with 80–87% of the sulfuric acid removed without losing sugars (<1%) and with a faradic yield of about 70–80% in 7–20 min [71,92].

### 3.8. Combination of Different Techniques

The technologies mentioned previously have sometimes been combined in integrated purification processes of lignocellulosic acid extracts.

For instance, a monomeric sugar acid hydrolysate obtained from bamboo was treated by AC to remove color compounds (e.g., phenolic compounds, furanic compounds), then by SMB, to separate the sulfuric acid from the sugars. Under the best conditions, AC led to the removal of 93% of the color with no adsorption of sugars, and SMB led to the recovery of 90.5% of the sulfuric acid and the acetic acid on one side, and of 99.9% of xylose and 97.4% of glucose on the other side [67].

Another combination of different purification techniques involved the use of ultrafiltration to remove macromolecules such as lignin or proteins from a wheat bran acid extract, then ED to recover sulfuric acid, and finally, ion exchange to complete the demineralization (conductivity < 10 µS/cm) [71,92]. Overall, the sugars’ recovery was 90% and their purity close to 100%.

## 4. Conclusions

The best yields for the conversion of cellulose and hemicelluloses into monomeric sugars were obtained with a combination of hydrolyses: one at a high concentration of sulfuric acid (about 70% (*w*/*w*)) at a low temperature (about 30 °C) followed by another step at a low concentration of sulfuric acid (about 4% (*w*/*w*)) at a high temperature (about 120 °C). These fractionation conditions produced an extract that contained all the sugars under their monomeric form, but also mineral acid, acetic acid, furans, and phenolic compounds. Purification of the sugars implies removing the fermentation inhibitors and the mineral acid to potentially recycle it. Adsorption or membrane filtration were efficient techniques to remove fermentation inhibitors. The removal of the mineral acid was already carried out industrially using low pressure chromatography on strong acid cation-exchange resin with water as the eluent. Once the purified extract contained only monomeric sugars, fermentation could be done directly on the sugar mixture with the appropriate enzymes for the production of ethanol for example, or sugar/sugar separation can be carried out by another step of low pressure chromatography before further valorization of the individual sugars into more valuable chemicals.

## Figures and Tables

**Table 1:** Yields of sugar monomers produced after treatment on lignocellulosic biomass under optimized dilute acid conditions. S:L, solid:liquid; DS, dry solid.

Biomass	Variable	Optimized Conditions	Monomer Yield	Reference
Sugarcane bagasse	0.25–7% H_2_SO_4_ (*v*/*v*) No variation of S:L ratio No variation of temperature Duration: 15–240 min	0.5% H_2_SO_4_ (*v*/*v*) S:L ratio 1:15 (*w*/*v*) 121 °C 60 min	44% glucose 74% hemicelluloses	[49]
Sugarcane bagasse	0–3% H_2_SO_4_ (*w*/*v*) No variation of S:L ratio 112.5–157.5 °C 5–35 min	1.5% H_2_SO_4_ (*w*/*v*) S:L ratio 1:6.7 (*w*/*v*) 135 °C 20 min	62% xylose	[59]
Sugarcane bagasse	0.25–8% H_2_SO_4_ (*w*/*w*) S:L ratio 1:5–1:20 (*w*/*w*) DS 80–200 °C 10–2000 min	4% H_2_SO_4_ (*w*/*v*) S:L ratio 1:20 120 °C 60 min	80% xylose	[37]
Sugarcane bagasse	2–6% H_2_SO_4_ (*w*/*w*) No variation of S:L ratio 100–128 °C 0–300 min	2% H_2_SO_4_ (*w*/*w*) S:L ratio 1:10 (*w*/*w*) 122 °C 24.1 min	5% glucose 92% xylose	[32]
Sugarcane bagasse	2–6% HCl (*w*/*w*) No variation of S:L ratio 100–128 °C 0–300 min	2% HCl (*w*/*w*) S:L ratio 1:10 (*w*/*w*) 128 °C 51.1 min	8% glucose 100% xylose 36% arabinose	[33]
Sugarcane bagasse	2–6% HNO_3_ (*w*/*w*) No variation of S:L ratio 100–128 °C 0–300 min	6% HNO_3_ (*w*/*w*) S:L ratio 1:10 (*w*/*w*) DS 122 °C 9.3 min	7% glucose 85% xylose 32% arabinose	[34]
Sugarcane bagasse	2–6% H_3_PO_4_ (*w*/*w*) No variation of S:L ratio 100–128 °C 0–300 min	4% H_3_PO_4_ (*w*/*w*) S:L ratio 1:8 (*w*/*w*) DS 122 °C 300 min	6% glucose 60% xylose 33% arabinose	[35]
Rye straw	0.6–1.5% H_2_SO_4_ (*w*/*w*) No variation of S:L ratio No variation of temperature 30–90 min	1.5% H_2_SO_4_ (*w*/*w*) S:L ratio 1:10 (*w*/*v*) DS 121 °C 90 min	10% glucose 65% xylose 67% arabinose	[52]
Bermudagrass	0.6–1.5% H_2_SO_4_ (*w*/*w*) No variation of S:L ratio No variation of temperature 30–90 min	1.5% H_2_SO_4_ (*w*/*w*) S:L ratio 1:10 (*w*/*v*) DS 121 °C 90 min	33% glucose 59% xylose 65% arabinose	[52]
Sweet sorghum bagasse	No variation of acid concentration No variation of S:L ratio No variation of temperature No variation of duration	0.5% H_2_SO_4_ (*w*/*w*) S:L ratio 1:20 (*w*/*v*) 170 °C 20 min	85% xylose	[60]

For SCB, glucose was supposed to come from cellulose exclusively, and xylose and arabinose were supposed to be the only components of the hemicelluloses.

**Table 2:** Dilute acid pretreatment of corn stover - hydrolysis reactions and assumed conversions based on the NREL technical report NREL/TP-5100-60223 (2013) [19].

Reaction	Reactant	% Converted to Product
(Glucan)_n_ + H_2_O → n Glucose	Glucan	9.9%
(Glucan)_n_ + H_2_O → n Glucose Oligomer ^a^	Glucan	0.3%
(Glucan)_n_ → n HMF + 2n H_2_O	Glucan	0.3%
Sucrose → HMF + Glucose + 2 H_2_O	Sucrose	100%
(Xylan)_n_ + n H_2_O → n Xylose	Xylan	90.0%
(Xylan)_n_ + m H_2_O → m Xylose Oligomer ^a^	Xylan	2.4%
(Xylan)_n_ → n Furfural + 2n H_2_O	Xylan	5.0%
Acetate → Acetic Acid	Acetate	100%
(Lignin)_n_ → n Soluble Lignin	Lignin	5.0%

^a^ Sugar oligomers are considered soluble, but not fermentable.
